# The Cost of Voluntary Medical Male Circumcision in South Africa

**DOI:** 10.1371/journal.pone.0160207

**Published:** 2016-10-26

**Authors:** Michel Tchuenche, Eurica Palmer, Vibhuti Haté, Ananthy Thambinayagam, Dayanund Loykissoonlal, Emmanuel Njeuhmeli, Steven Forsythe

**Affiliations:** 1 Health Policy Project, Project SOAR (Supporting Operational AIDS Research), Avenir Health, Washington, District of Columbia, United States of America; 2 Health Policy Project, Palladium Consultant, Johannesburg, South Africa; 3 George Washington University, Washington, District of Columbia, United States of America; 4 USAID, Pretoria, South Africa; 5 National Department of Health, Pretoria, South Africa; 6 USAID, Washington, District of Columbia, United States of America; University of Ottawa, CANADA

## Abstract

Given compelling evidence associating voluntary medical male circumcision (VMMC) with men’s reduced HIV acquisition through heterosexual intercourse, South Africa in 2010 began scaling up VMMC. To project the resources needed to complete 4.3 million circumcisions between 2010 and 2016, we (1) estimated the unit cost to provide VMMC; (2) assessed cost drivers and cost variances across eight provinces and VMMC service delivery modes; and (3) evaluated the costs associated with mobilize and motivate men and boys to access VMMC services. Cost data were systematically collected and analyzed using a provider’s perspective from 33 Government and PEPFAR-supported (U.S. President's Emergency Plan for AIDS Relief) urban, rural, and peri-urban VMMC facilities. The cost per circumcision performed in 2014 was US$132 (R1,431): higher in public hospitals (US$158 [R1,710]) than in health centers and clinics (US$121 [R1,309]). There was no substantial difference between the cost at fixed circumcision sites and fixed sites that also offer outreach services. Direct labor costs could be reduced by 17% with task shifting from doctors to professional nurses; this could have saved as much as $15 million (R163.20 million) in 2015, when the goal was 1.6 million circumcisions. About $14.2 million (R154 million) was spent on medical male circumcision demand creation in South Africa in 2014—primarily on personnel, including community mobilizers (36%), and on small and mass media promotions (35%). Calculating the unit cost of VMMC demand creation was daunting, because data on the denominator (number of people reached with demand creation messages or number of people seeking VMMC as a result of demand creation) were not available. Because there are no “dose-response” data on demand creation ($X in demand creation will result in an additional Z% increase in VMMC clients), research is needed to determine the appropriate amount and allocation of demand creation resources.

## Introduction

The first HIV diagnosis was in South Africa in 1983 [1]. Now, South Africa has the highest number of people living with HIV of any country in the world: 6.8 million people, or 12.96% of the total population [2]. Key drivers of the epidemic are intergenerational sex, multiple concurrent partners, low condom use, low rates of male circumcision, and gender inequality [3–5].

Medical male circumcision has been shown to be a promising method and one of the most cost-effective of those available for preventing new HIV infections [6,7], with greater benefits for men in the general population if implemented in conjunction with other evidence-based prevention methods [8] such as condom use and pre-exposure prophylaxis and therapeutic measures such as antiretroviral treatment of the HIV-positive partner [9]. The protective effect of voluntary medical male circumcision (VMMC) against sexually transmitted diseases has been well-documented [10,11], and integration with existing HIV prevention strategies is expected to maximize benefits both for men and women [3, 12]. There is strong evidence from observational data and three randomized controlled trials that medically circumcising men lowers their risk of acquiring HIV through heterosexual intercourse by between 38% and 66% [13–15, 7, 8, 10]. In turn, as a result, VMMC also provides long-term indirect protection to women [3, 12]. This success led the Joint United Nations Programme on HIV/AIDS (UNAIDS) and the World Health Organization (WHO) to identify 14 priority countries in eastern and southern Africa (including South Africa) for VMMC scale-up [16, 17]. South Africa has a very low uptake of VMMC, so on the basis of this overwhelming evidence, in 2010 the South African Government introduced the procedure as an HIV prevention intervention [18]. Evidence shows that VMMC also has a population-level effect and can substantially reduce HIV incidence [19, 20]. Additional studies found that VMMC offers durable protection, with prevention benefits documented five years after VMMC [21, 22].

It is currently estimated that 46.4% of all males over age 15 in South Africa have been circumcised, through either a traditional or a medical procedure. However, only 18.6% of males have been circumcised medically [12, 23]. The ultimate goal of the South African Government’s VMMC program is to reduce HIV incidence, by scaling up medical male circumcision (MMC) to reach 80% of HIV-negative males 15–49 years old by 2016 [24]. This could avert more than 1 million new HIV infections through 2015 [25, 6]. This benefit (the 1 million HIV infections averted) is solely attributable to the MMCs.

South Africa established the most ambitious and largest target in the 14 priority countries: to circumcise 4.3 million men and boys between 2010 and 2016. By early 2015, the South African VMMC program had performed approximately 1.8 million VMMCs [26], or 43% of its target.

Three previous costing studies have attempted to assess the unit cost of male circumcision in South Africa. These studies (having unique advantages as well as some limitations) were conducted by the U.S. Agency for International Development (USAID)-funded Health Policy Initiative (HPI); Optimizing the Response in Prevention: HIV Efficiency in Africa (ORPHEA); and the Clinton Health Access Initiative (CHAI). The objectives of these costing studies were to assess the resources required for VMMC scale-up and to identify opportunities for potential cost savings. The HPI costing study was conducted in 2008, before South Africa introduced VMMC as an HIV prevention strategy. This study estimated that the mean cost per circumcision, based on nine costed sites, was $49 (R525) [27]. All dollar figures throughout are U.S. currency. The ORPHEA study, based on 27 sites, was conducted in 2012 and estimated a unit cost of $135 (R1,460) per circumcision [28]. The CHAI study, concluded in 2015, estimated that the unit cost per circumcision performed was $144 (R1,561) [24]. Most limitations of these previous studies have been addressed herein. The ORPHEA study was conducted in three out of the nine provinces of South Africa, while this study includes data from eight of those provinces. Moreover, this study constitutes a detailed economic analysis that considered all costs—direct and indirect—of continuous quality improvement (CQI) of VMMC services, a component that was not included in the ORPHEA analysis. Also, the present study considers which modes of service delivery are the most efficient and the geographic location of sites (urban versus rural), whereas the CHAI study considered cash flows in a specific period, predominantly in order to budget future resource needs. Given South Africa’s very ambitious target in terms of the number of circumcisions to be completed to reach 80% VMMC coverage among adult men, the unit cost obtained from our study can inform the scale-up of VMMC in South Africa.

The study objectives are to derive the unit cost of delivering VMMC in South Africa at the facility level and to identify the level of spending currently incurred for VMMC demand creation.

## Methods

### Ethical Considerations

The Human Research Ethics Committee (Medical) of the University of the Witwatersrand, South Africa, and the Health Media Lab’s Institutional Review Board, in Washington DC—which is authorized by the U.S. Department of Health and Human Services (DHHS), Office of Human Research Protections, and which has DHHS Federal-Wide Assurance approval—provided ethical approvals for this costing study.

### Data Collection

This study initially selected three sites randomly (from a list of approximately 900 sites offering VMMC services in South Africa) in each of the nine provinces to be costed. However, some of these sites were no longer operational. Other VMMC programs were operating services out of schools, which would have required approval from South Africa’s Department of Basic Education in order to conduct the costing. Thus, through an extensive consultative stakeholder engagement process, which was necessary to obtain provincial buy-in and support for the study, facility data collection was conducted at 33 sites (see S1 Table) across eight of the nine provinces in South Africa. Selection criteria were geographic area in the province (for example, urban, peri-urban, or rural sites) and mode of service delivery (for example, fixed only sites versus fixed sites with outreach services).

The facility data collection form was based on similar structured questionnaires developed to cost male circumcision in Tanzania [29] and Kenya [30]. A provider’s perspective was adopted for the analysis. Before data collection commenced, a series of activities was conducted to evaluate the study instrument. This included pretesting the instrument at a facility in Gauteng. Cost data from facilities were retrospectively collected for 12 months (for most facilities, this was January to December, 2014). Interviews were semi-structured and directed at key personnel at the 33 selected sites.

#### Data Sources

Data were collected from relevant sources at the facilities, including outpatient registers, pharmacy registers, maintenance departments, and laboratory departments. Data not available from these facilities were obtained from the district, provincial, or national levels within the Department of Health or from the U.S. President’s Emergency Plan for AIDS Relief (PEPFAR) implementing partners if items had been purchased by these organizations. Human resources, financial, and utilization data were gathered from official records from both facilities and PEPFAR implementing partners. However, VMMC program management and overhead costs of partner organizations and government were not included in this study. Costs of training, CQI, and demand creation were obtained directly from South Africa’s PEPFAR implementing partners. Unit costs were derived using an ingredient-based approach, where all inputs are listed, their costs are collected, and the contribution of these costs to the overall cost is quantified [29]. A top-down approach could have been performed by assessing the resources that are allocated to each facility. Generally the top-down approach is easier to perform, because it does not require direct interviews with facilities. On the other hand, the ingredient-based approach tends to collect a greater depth of cost and effectiveness data, as information is collected directly through interviews with the facilities.

Major cost categories in the unit cost calculation were

- Direct costs (drugs and supplies, other consumables, equipment, non-consumable supplies, and direct and indirect personnel)
- Indirect costs (capital, maintenance, electricity and fuel, other utilities, support personnel, and management and supervision)

Overhead costs (such as water, electricity, Internet, telephone, waste management, cleaning services, etc.) and the rental or construction value of a facility were assigned to the VMMC program either by using the annual rental value of the entire facility or by identifying the original construction value of the facility. If facilities were able to provide a rental value, costs were allocated to the MMC program based on the proportion of the total facility space used for circumcisions. At each facility, costs related both to direct and indirect staff were collected. Direct staff largely were clinical staff, such as general practitioners, clinical associates, and nurses. Indirect staff were individuals employed by a site to provide overall facility support, but who generally were not working exclusively on the VMMC program. These might include, for example, security guards, maintenance staff, facility managers, office assistants, receptionists, and drivers. These indirect staff members often were compensated by the facility itself and were typically not paid through the VMMC program budget. Information on employment status (permanent versus contracted staff) was collected both for direct and indirect staff members. Additional information collected includes the number of personnel, salaries, and the percentage of time allocated to VMMC. Where external staff were introduced to a National Department of Health- or PEPFAR-supported site, information on salaries was collected from the implementing partners who hired the external staff. In some cases, implementing partners provided “roving teams” who traveled to sites once or twice a week. In these cases, the proportion of time spent at a site was estimated and used to allocate cost to each of the relevant sites.

In situations where indirect staff members were not employed predominantly by the VMMC program, an allocation method was developed such that the proportion of VMMC clients relative to the total client volume at the facility was used to approximate indirect staff time to VMMC costs. For instance, if VMMC represented 5% of all clients at the facility, then 5% of the salaries of guards, maintenance staff, and others were allocated to the VMMC program. Detailed information regarding the method for collecting cost data for circumcision kits, medications and other consumables, equipment and furniture, vehicles, and overhead for continuous quality improvement is provided in S1 Text.

Although data on total demand creation spending was collected, this was not translated into a demand creation unit cost owing to the complexity of linking spending on demand creation to a specific output (e.g., additional VMMC clients). As a result, demand creation costs were not included in the calculation of unit cost per person assessing VMMC services in South Africa.

Finally, where cost data were compared for statistical purposes (e.g., comparing unit costs at fixed only sites versus fixed sites with outreach services), a Student’s *t*-test was performed using Excel’s statistical functions. Independent *t*-test with unequal variance is often used to determine if two sets of data (e.g., fixed sites with no outreach services and fixed sites with an outreach component) are significantly different from each other. The assumption of unequal variance is used to account for the possibility that the samples may be skewed [31].

### Costing Model

The data were entered directly in the developed costing model: an Excel spreadsheet customized to calculate only as key output the unit cost of VMMC by mode of service delivery in South Africa [32].

## Results

The costing activity included initial collection of financial and human resource data required for estimating unit cost per VMMC beneficiary. Unit costs were calculated from an ingredients-based approach by mode of service delivery (fixed versus fixed with outreach services); cost drivers (direct labor, consumables, CQI, indirect labor, overhead, training, equipment, and vehicles); province; level of urbanization (urban, peri-urban, or rural); scale of operations; and type of facility where the services were performed (hospital versus healthcare center/clinic). In this ingredients-based approach, the system’s elements first are listed and then their individual costs are collected.

### Cost of Service Delivery

At the average exchange rate for 2014 of R10.83 = $1, the unit cost at the 33 facilities was determined to be $132 (R1,431) per circumcision performed. The most common modes of service delivery are: (1) fixed (static) sites; (2) fixed with outreach services; and (3) mobile services. The focus of this analysis was on the comparison of unit costs for fixed sites versus those fixed sites that also had outreach services (mobile sites were not available for costing in any of the provinces). A total of 25 sites were fixed only and eight sites were fixed with an outreach component.

### Cost by Type of Facility

**Fig 1:**
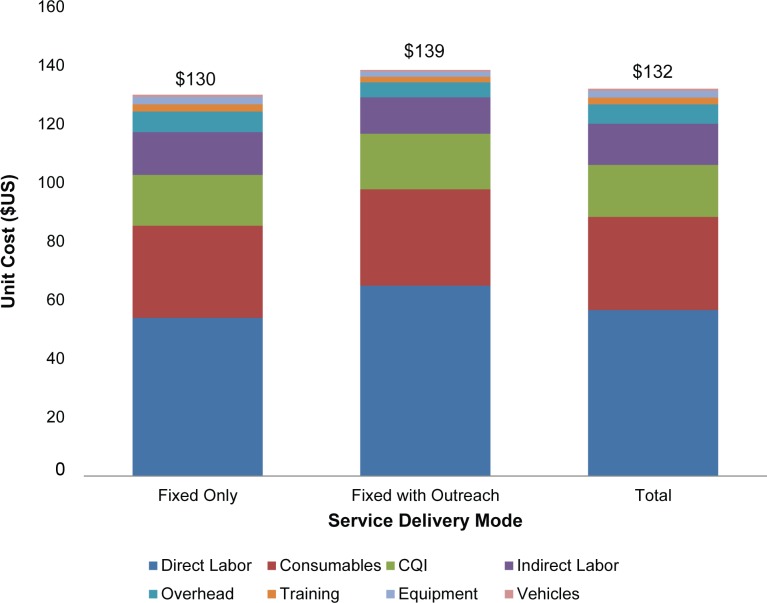
Unit costs by mode of service delivery.

Fig 1 shows the difference between fixed sites (no outreach services) versus those that have both fixed and outreach components. The unit cost for sites with outreach services with mean M = $138.50 (R1,500) and standard deviation SD = $15.70 (R170) was slightly higher than for sites without outreach services: M = $130.10 (R1,409), SD = $8.22 (R89), although this difference was not statistically significant (*p* = 0.322). Several reasons might explain why unit costs do not appear to differ significantly. First, sites with outreach services may enjoy economies of scale that counteract the additional costs associated with transporting consumables, equipment, and staff to communities. Facilities with outreach services had an average of 3,348 circumcisions per year, whereas facilities without outreach services had an average of only 2,128 circumcisions per year, indicating that sites with outreach services did enjoy economies of scale. In addition, fixed sites might also incur additional costs associated with bringing clients to their facilities, whereas sites with outreach services might not require, for example, transport provided to clients.

Fig 1 also indicates the components of the unit costs. The largest of these is direct labor, which represents 43% of all costs. This is followed by consumables, which represent 24% of all costs. This result is in agreement with earlier studies, which found that the two main cost drivers associated with providing VMMC in sub-Saharan Africa are personnel and consumables [33,34]. The consumables include the cost of the male circumcision kit, which is the most expensive component of the consumables costs. The next most expensive cost component is CQI, which represents 13% of all costs. CQI costs are included here, because high-quality services increase client satisfaction and potentially generate more demand for VMMC. The next cost category is indirect labor, which accounts for 11% of all costs. The remaining 9% of costs is represented by overhead expenses, training, equipment, and vehicles.

### Cost by Province

**Fig 2:**
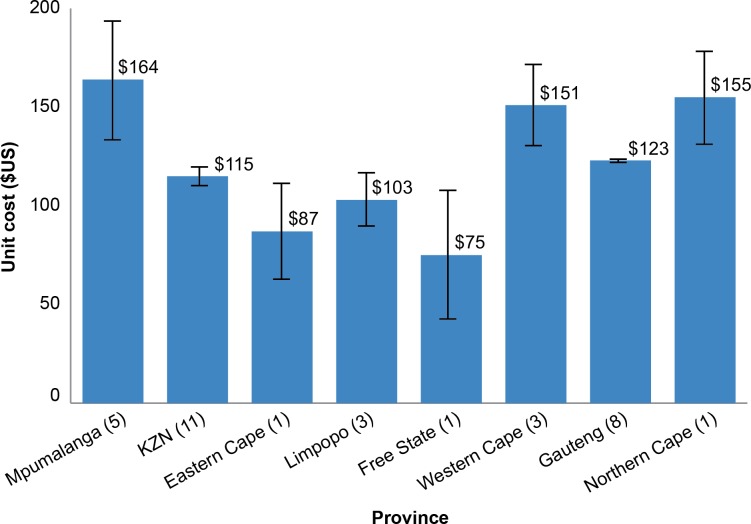
Unit cost by province.

Fig 2 shows a breakdown of unit costs by province. The number of VMMC sites within a given province is indicated in parentheses next to the province name. The largest number of sites in this analysis is located in KwaZulu-Natal (11 sites), followed by Gauteng (8 sites). In both provinces, the unit cost did not differ substantially from the overall unit cost. Mpumalanga province has the highest unit cost by province. All five sites within Mpumalanga have consistently high unit costs for labor and medicines/consumables. It is interesting to note that three out of the five facilities in Mpumalanga have very high proportions of contracted labor in their clinical labor force, compared to other sites that have an equal or higher proportion of permanent labor. This might indicate that permanent clinical staff can be acquired at more competitive prices than contractual clinical staff. This is especially important in the case of essential clinical labor, such as general practitioners, clinical associates, and professional nurses—commonly high-cost human resources. The least expensive province was Free State, although this province was represented by only one site.

### Cost by Urbanization Level

**Fig 3:**
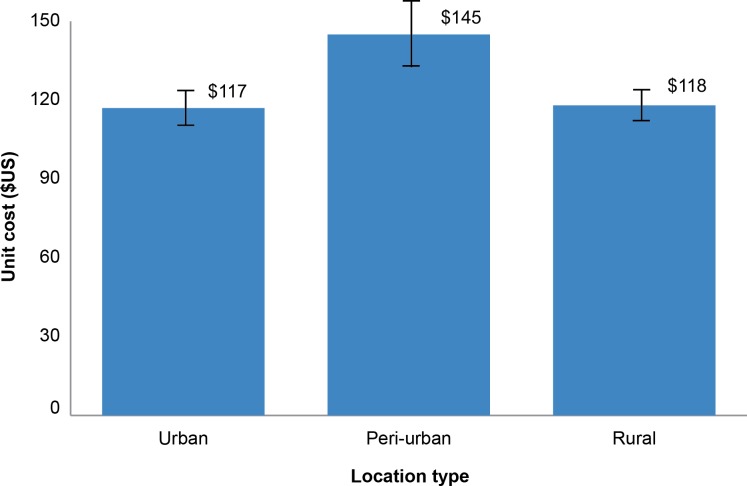
Unit cost by province urbanization level.

Each site was classified as being located in urban, peri-urban, or rural areas of the country. Fig 3 illustrates how unit costs differ depending on the level of urbanization in the community where the facility operates. There were 13 urban sites, 10 peri-urban sites, and 10 rural sites. Fig 3 indicates that the unit cost is unrelated to the level of urbanization of the site. In fact, urban and rural sites appear to have almost identical unit costs, $117 (R1,270) and $118 (R1,279), respectively. The higher unit cost in peri-urban sites $145 (R1,565) appears to be driven by two sites with high unit costs.

### Cost by Scale of Operation

**Fig 4:**
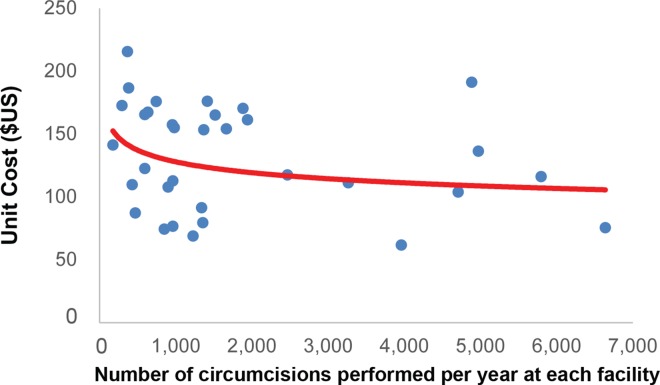
Unit cost by facility scale of operations. The red line indicates the relationship between the VMMC unit cost at the facility and the scale of the operation (circumcisions performed per year at each facility).

Fig 4 shows the relationship between the number of circumcisions performed in the past 12 months and the average unit cost of each circumcision. On the one hand, about 45% of all sites had fewer than 1,000 circumcisions per year. The average unit cost of these lower-volume facilities was $136 (R1,473). On the other hand, about 21% of all sites reported more than 3,000 circumcisions per year. These higher-volume sites had a unit cost of $114 (R1,231). The red line in Fig 4 shows the calculated relationship between the VMMC unit cost at the facility and the scale of the operation (circumcisions performed per year at each facility). As expected, there is an inverse relationship between volume and unit cost: sites with higher numbers of VMMC clients generally have a lower unit cost. However, scale does not fully explain the variation in unit cost. Some lower-volume sites, for example, also have a low unit cost, and some higher-volume sites have a high unit cost.

### Unit Cost by Type of Health Facility

**Fig 5:**
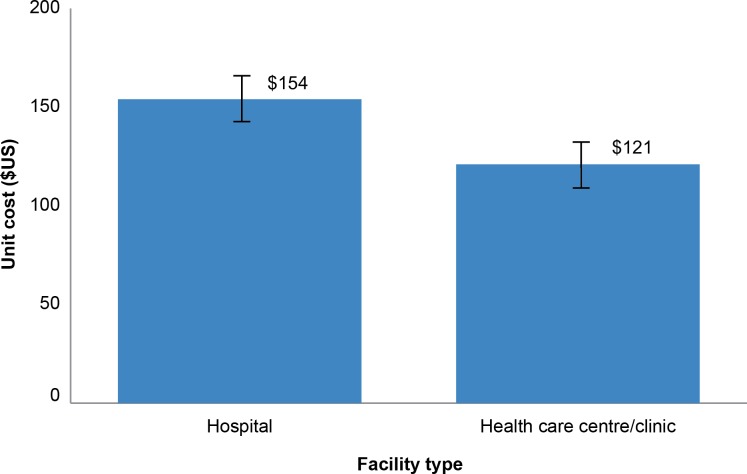
Unit cost at hospitals versus healthcare centers/clinics.

Of the 33 sites where unit cost data were collected, 11 were hospitals and 22 were health centers/clinics. As Fig 5 shows, the unit cost at hospitals ($154 [R1,666]; M = $154 [R1,666], SD = $9.41 [R102]), is higher than at health centers/clinics ($121 [R1,313]; M = $121.24 [R1,313], SD = $9.05 [R98]). The difference in this case is statistically significant (*p* = 0.009). Most of the differences are attributable to higher labor costs (both direct and indirect) at hospitals than at health centers and clinics.

### Task Shifting’s Impact on Labor Costs

Task shifting—the planned delegation of tasks from higher level health cadres (specialists or doctors) to non-physician clinicians [35]—has been proposed as a way to expand surgical (human resource) capacity, particularly in resource-limited settings [36]. It has long been promoted by WHO as a potential solution to expanding VMMC services [37], by increasing the efficiency and effectiveness of services recommended to implement the models for optimizing volume and efficiency [23]. To assess the potential cost savings of task shifting, an analysis was performed in which the salaries of doctors and clinical associates were replaced by the salaries of professional nurses.

**Fig 6:**
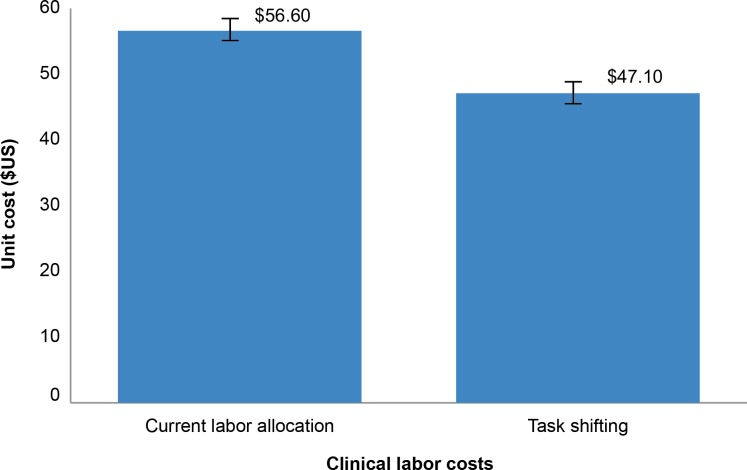
Potential impact of task shifting on clinical labor costs.

Fig 6 shows the direct labor costs with and without task shifting. The current direct labor unit costs for male circumcision are estimated to be $56.60 (R613). However, if the doctors were replaced by professional nurses, this could be reduced to $47.18 (R511) per circumcision, a saving of $9.41 (R102), representing 17% of direct labor costs (or 7% of the unit cost per circumcision). The Government of South Africa established a target of performing 1.6 million circumcisions in 2015. Thus, if the average cost savings from task shifting to nurses were achieved, the total savings in 2015 alone would have been $15 million (R163.20 million). This saving estimate does not include the cost of training the additional nurses; if training is considered, this savings could be reduced slightly.

Bear in mind that this calculation might not reflect all of task shifting’s advantages. Most notably, nurses are much more widely available in health facilities than doctors and clinical associates are. Because VMMC services often are not offered when a doctor is unavailable, task shifting circumcisions to nurses not only might lower costs but also might permit a much larger number of circumcisions to be performed. There are also opportunities for cost savings in integrated primary care settings, where facility costs and staff are shared across multiple activities.

Furthermore, data from the systematic monitoring of the VMMC scale-up in an eastern and southern Africa study [38] indicate that doctors are much more likely to burn out and leave the VMMC program, whereas nurses report higher levels of job satisfaction, and therefore are more likely to continue providing VMMC services long after their training has ended. Thus, nurses require less retraining costs than doctors. Consequently, due to reduced staff turnover, task shifting could also improve VMMC program quality.

### Cost of Demand Creation

Demand creation—mobilizing and motivating men to access VMMC services [39]—is crucial for the successful implementation of a VMMC program. Information about South Africa’s VMMC spending on demand creation was obtained from PEPFAR’s VMMC demand creation data and communication with implementing partners. In addition, 2014 business plans of South Africa’s nine provinces were reviewed to extract relevant VMMC demand creation budgetary data. These business plans produced limited cost data, because most provincial plans do not contain specific line items for a comprehensive VMMC demand creation picture. Finally, about a quarter of the facilities (8 out of 33) provided VMMC demand creation information. The cost data from these facilities were then extrapolated nationally to the approximately 900 sites where VMMC services are available in South Africa.

Actual spending on VMMC demand creation (including community mobilization channels and mass and small media costs) were obtained directly from the following PEPFAR implementing partners supporting the national, regional, and local VMMC communication and demand creation strategies: Anova Health Institute; Aurum Institute; CareWorks; Centre for HIV/AIDS Prevention Studies; Community Media Trust; Johns Hopkins Health and Education South Africa; Jhpiego (an affiliate of Johns Hopkins University); Right to Care; Southern African Clothing and Textile Workers’ Union Worker Health Program; Society for Family Health; and TB/HIV Care Association. No information on the cost of VMMC demand creation was collected at the national government level, because the National Department of Health does not conduct VMMC demand creation activities.

**Fig 7:**
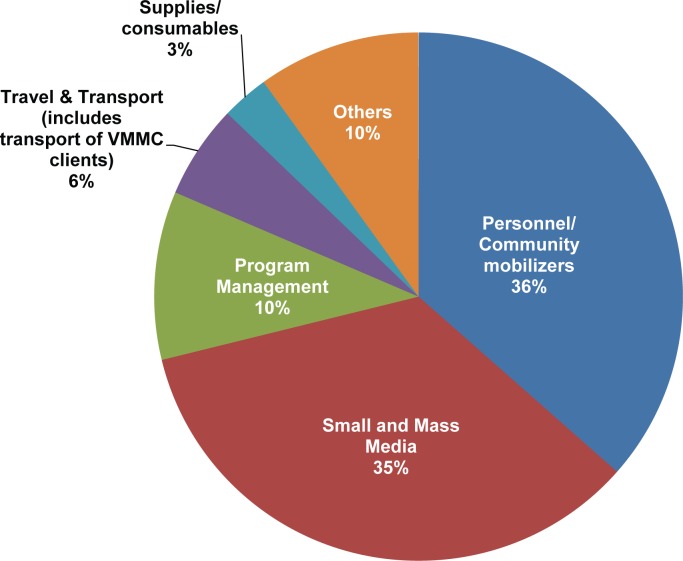
Demand creation by major cost category.

Approximately $14.2 million (R154 million)—including funding from PEPFAR and other sources—was reported to have been spent on VMMC demand creation over the most recent 12-month period (January to December 2014), of which $13.5 million (R146.2 million) was reported by PEPFAR implementing partners. The latter was disaggregated into major cost categories, as shown in Fig 7. Data on the remaining $461,000 (R5 million), which could not be disaggregated into cost categories and therefore was not included in Fig 7, were derived both from the provincial business plans—approximately $92,000 (R1 million)—and nationally extrapolated data from facilities that reported expenditures on VMMC demand creation of $369,000 (R4 million).

PEPFAR fiscal year 2014 total expenditures on VMMC activities in South Africa were $54.2 million (R587 million) [40]. Thus, the 2014 VMMC demand creation expenditures represent about 26% of all resources spent by PEPFAR on male circumcision in South Africa.

Major VMMC demand creation cost drivers are interpersonal communication (personnel and community mobilizers, 36%); mass and small media (35%); demand creation program management (10%); travel and transport, including transport of VMMC clients (6%); and supplies and consumables (3%). The “other” category—which includes furniture and equipment, systems development, applied research, demand creation training, and any other VMMC communication channels (phone messaging/mobile signage, and data collection and reporting)—represents 10% of the total VMMC demand creation cost.

## Study Limitations

While efforts were made to produce a comprehensive analysis of costs, this study does not purport to be exhaustive. A number of limitations should be noted.

First, the relatively small sample size for the facility survey (33 sites, representing 3.8% of the total VMMC sites from eight of the nine provinces) makes it difficult to extrapolate costs to the national program. In addition, despite all efforts to collect data from all of the nine provinces in the country, data from North West province could not be accessed, because of delays in obtaining approvals and the subsequent incompleteness of the data. A larger sample size might have provided better opportunities to compare unit costs across provinces.

Second, the exclusion of mobile sites is a limitation of this study in terms of estimating costs by mode of service delivery. Including mobile sites would have provided greater clarity regarding the relative costs of the three main modes of service delivery.

Third, the lack of data from the private sector represents another gap in knowledge concerning the overall cost of scaling up services in South Africa. The National Department of Health is increasingly interested in using general practitioners to expand coverage, either within private or public sector facilities. The National Department of Health and PEPFAR have prioritized an additional data collection effort that will address this limitation. A follow-up study to estimate the private-sector unit cost of providing VMMC services is in progress, and it is expected that the results will help to fill this knowledge gap.

Fourth, efforts were made to obtain all information about resources used at the facilities, with the interview process requiring respondents to accurately recall and identify resources directly and indirectly used by the facility. However, no time-motion analysis was carried out to assess how staff spent their time on the VMMC program. Instead, respondents were asked to provide a general allocation of time during each day. Because the time allocation was based on recall and not on actual observations, there may have been an overestimation or underestimation of time spent on male circumcision. In addition, this study did not identify how unit costs might change in the future as South Africa further scales-up its VMMC program. On the one hand, unit costs might decline as a critical mass of clients is reached. On the other hand, unit costs might also increase as identifying clients who are ready and willing to undergo male circumcision becomes increasingly difficult.

Fifth, CQI and training costs include a mix of variable and fixed costs, and consequently this study could not categorized costs into fixed versus variable costs.

Finally, this study assessed current demand creation spending and did not determine the ideal levels of spending or ways to strengthen demand creation to increase VMMC service delivery. Cost data were collected on actual VMMC demand creation spending and not on the current need for demand creation activities. Estimating the unit cost of demand creation per person circumcised was not investigated, because the study did not capture any data on linking service provision to demand creation activities. For these reasons, the demand creation cost was not included in the unit cost per person assessing the VMMC service.

## Conclusion

The 2014 unit cost of VMMC in South Africa is estimated to be an average of $132 (R1,431). This estimate is higher than the first unit cost study in South Africa $49 (R525), which was based on data from 2008. However, the unit cost is very comparable to more recent estimates made as part of the ORPHEA study $135 (R1,460) [28]; and the CHAI analysis $144 (R1,561) [24]. Most of the three previous studies’ limitations have been addressed in the present study. The ORPHEA study was conducted in three out of the nine provinces of South Africa, while this study included data from eight provinces. This study is a detailed economic analysis that includes costs such as CQI, a component that was not included in the ORPHEA analysis. The CHAI study took a predominantly top-down approach, whereas this study focused predominantly on data collection using an ingredient-based approach, where each resource required for the particular intervention being studied is identified and valued.

The largest component of the VMMC unit cost in the present study is direct labor, which accounts for 43% of all costs. This is followed by consumables (24%), CQI (13%), and indirect labor (11%). The fact that direct labor accounts for such a large proportion of the overall costs indicates that any attempt to reduce unit costs would need to focus on these large cost items.

One noted area of costs savings could potentially be realized through task shifting. Task shifting is projected to reduce the direct labor costs by 17%: $9.41 (R102). By lowering service delivery costs, this allows for existing resources to be extended further. Given South Africa’s target of 1.6 million circumcisions in 2015, task shifting could have saved $15 million (R163.20 million) in 2015 alone. This figure could be slightly lower if the cost of training the required number of additional nurses is taken into account. However, the benefits of task shifting are likely to exceed merely the costs saved. Task shifting has the potential to increase the number of circumcisions that are performed and reduce retraining costs (because doctors remain in their current practice for a shorter time than nurses do). Another approach in which the cost of VMMC could be reduced is by focusing on high-volume sites, because these high-volume sites generally incur lower per-unit costs.

Finally, demand creation for VMMC warrants further analysis. This study found that $14.2 million (R154 million) from PEPFAR and other funding sources was spent on VMMC demand creation in 2014. Most of these resources are spent by PEPFAR implementing partners, although resources are also being spent by provinces and some facilities. A large proportion of these resources are for personnel, including community mobilizers (36%), and for promotion using mass and small media outlets (35%). The government’s ambitious target of producing 4.3 million circumcisions through 2016 requires a tremendous amount of demand creation work, but there is uncertainty about the level of demand creation that would be required in order to reach this goal, and there are currently no VMMC program data to guide the government and PEPFAR implementing partners toward a better allocation of VMMC demand-creation resources.

The future cost of circumcision kits over the short and long term may potentially influence the estimates of future circumcision cost. Additional items to be included in the kit—such as dissecting scissors and a marking pen that will be used for the dorsal slit method—are likely to increase the cost of these kits from $15 (R162.45) to $22 (R238.26). However, as more kits are purchased, economies of scale will likely bring the cost of the kits down.

This study focused only on the cost of surgical circumcision. However, South Africa is currently testing the feasibility of introducing PrePex,^TM^ an adult disposable medical device developed in 2009 to facilitate nonsurgical VMMC. This elastic ring controlled radial compression device causes necrosis of the foreskin in at most seven days. VMMCs conducted using the PrePex^TM^ device does not require anesthesia or suturing [41] and a nurse can administer the procedure. An analysis of the cost of adding PrePex^TM^ device-based circumcisions to an existing surgical VMMC program, as part of a PrePex^TM^ demonstration study, is being finalized in South Africa, Tanzania, and Lesotho.

## Supporting Information

S1 Text Additional Details on How Other Costs Were Collected. (DOCX)

S1 Table VMMC Facility Survey Sites. (DOCX)
