# Correction: The Cost of Voluntary Medical Male Circumcision in South Africa

**DOI:** 10.1371/journal.pone.0169710

**Published:** 2017-01-03

**Authors:** Michel Tchuenche, Eurica Palmer, Vibhuti Haté, Ananthy Thambinayagam, Dayanund Loykissoonlal, Emmanuel Njeuhmeli, Steven Forsythe

There is an error in the Funding statement. The number for the Cooperative Agreement Project SOAR (Supporting Operational AIDS Research) is incorrect. The correct number is OAA-A-14-00060.
