# Supplementary material for: The Cost of Voluntary Medical Male Circumcision in South Africa
Source: PLoS One. 2016 Oct 26;11(10):e0160207. doi: 10.1371/journal.pone.0160207 (PMC5082632; doi:10.1371/journal.pone.0160207)
Supplement: S1 Text — (DOCX) [file pone.0160207.s001.docx]

**S1 TEXT. Additional Details on How Other Costs Were Collected**

**Circumcision Kits**

The cost of circumcision kits purchased in South Africa has varied significantly over time. When kits were first introduced in 2010, the cost was US$23 per kit. This cost has subsequently declined as bulk purchases have been made. Discussions with Supply Chain Management Systems (SCMS) have indicated that kits are currently being purchased in South Africa at a cost of US$13.05. The combined costs of technical assistance and supply chain management further increased the kit price to US$15.10. It should be noted, however, that there is some uncertainty regarding the future price of circumcision kits; while the cost of the circumcision kits is projected to rise in the immediate future to about US$22, due to the inclusion of new components to existing kits such as dissecting scissors, there may be significant declines in the medium to long term resulting from increased pressures to make kits more affordable.

**Medications and Other Consumables**

At each site, information was collected on the percentage of VMMC clients that received specific types of medicines/consumables, the quantity that they received, the input costs of each item, and the total calculated cost assigned to the facility for all VMMC clients. In most cases, facilities provided detailed information about the quantity distributed to each VMMC client. Input costs for medicines/consumables were collected from an array of sources. Priority was given to sites that provided their own estimates of input costs. However, it should be noted that in many cases, facilities were unaware of the purchase price of consumables since these items were typically either purchased at the national level by the National Department of Health (NDoH) or purchased by an NGO at the local level and delivered directly to the facility. In such cases, efforts were made to gather information from the NDoH or the NGO sources.

Input costs for a number of the medications and other consumables were collected from a variety of sources, including Supply Chain Management Systems (SCMS), the Clinton Health Access Initiative (CHAI), and PEPFAR implementing partners. Most notably, data on the input costs of medications were also obtained from Northdale Hospital—the VMMC Centre of Excellence in KwaZulu-Natal—which had extensive information on input costs for a number of items.

**Equipment and furniture**

Facilities were asked to (1) provide a list of all equipment and furniture utilized in the screening/review room, the counseling area, the operating theatre, and/or any relevant sterilization areas; and (2) identify general equipment that was used as part of the VMMC program. Each facility then provided information on the number of items utilized, the estimated percentage of time equipment/furniture was utilized by the VMMC program (in most cases, this was 100%, but in some cases equipment and furniture were shared with other programs within the facility), the replacement cost of each item, and the expected useful life of each item. Note that several furniture items, like desks, filing cabinets, etc., were originally purchased by the Government, the facility, or an implementing partner for use beyond the VMMC program.

To obtain the input cost of these items, facilities staff were asked to estimate the replacement cost of each item. In situations where they were unable to identify a replacement price, information was obtained from SCMS, CHAI, and/or PEPFAR implementing partners. In select cases where replacement costs were still not available, average costs were estimated based on the prices offered by South African private suppliers.

Useful life of equipment and furniture were mostly obtained from WHO Choice. When useful life estimates were not available from WHO CHOICE, data were collected from other circumcision costing studies performed in Lesotho and Tanzania.

**Vehicles**

Sites were also asked to (1) identify all vehicles used by the facility as part of the MC program; and (2) provide data on the number of vehicles, the replacement cost of the vehicles, and the proportion of time the vehicles were used by the VMMC program. While in select cases vehicles were used specifically for the purpose of the VMMC program, in most situations vehicles were used for general operational and programmatic purposes at the facilities and were occasionally used for the VMMC program. In the latter case, the proportion allocated to the VMMC program was determined based on the number of VMMC clients relative to the total number of clients at the facility as a whole.

Several facilities were unable to provide a replacement cost for vehicles. In these instances, on the basis of the information provided regarding the vehicles make, model, and year of manufacture, the costs of a comparable used vehicle were obtained through a popular South African used vehicles website.

**Overhead**

Annual overhead costs included a range of items, such as costs associated with utilities (water, electricity, internet, telephone, waste management, cleaning services, etc.) and the rental or construction value of a facility. Overhead costs were apportioned to the VMMC program based on the size of the space used for VMMC programmatic activities relative to the size of the entire facility. For instance, if circumcision services were offered within a 150-square-meter space inside a facility that was 1,500 square meters in size, then 10% of the costs of utilities would be allocated to the VMMC program.

Overhead costs related to the rental or construction value of the facility were assigned to the VMMC program either by determining the annual rental value of the entire facility or by identifying the original construction value of the facility. If facilities were able to provide a rental value, costs were allocated to the VMMC program based on the proportion of the total facility space used for circumcisions. However, in situations where the rental value was not available, the construction value of the facility was translated into a rental value by depreciating the construction value over a 40-year time period. In situations where neither the rental value nor the construction value could be obtained, it was assumed that the value of the facility was equivalent in rental terms to that of a median “typical” facility. In these cases, the rental value of the facility was determined to be US$3.80 per square meter.

**Continuous Quality Improvement**

To obtain estimates of the cost of Continuous Quality Improvement (CQI), information was obtained from the University Research Company (URC) in South Africa, which manages CQI for PEPFAR partners in South Africa. Of the 33 sites costed by this study, 27 were receiving CQI support from URC. Cost of CQI at each site included labor and travel costs, as well as costs associated with overhead, policy development, and so forth. The costs of the other 6 sites were estimated by URC based on the information available concerning adverse events, geographic location, and client volume at these facilities.

Facility data collection was ultimately conducted in a total of 33 sites across 8 different provinces of the country. The sites names are listed in Table S1.
