# Supplementary material for: The Cost of Voluntary Medical Male Circumcision in South Africa
Source: PLoS One. 2016 Oct 26;11(10):e0160207. doi: 10.1371/journal.pone.0160207 (PMC5082632; doi:10.1371/journal.pone.0160207)
Supplement: S1 Table — (DOCX) [file pone.0160207.s002.docx]

**Table S1. MMC Facility Survey Sites**

| **Province** | **Facility name** |
| --- | --- |
| Eastern Cape | Empilweni Community Health Centre |
| Free State | Lesedi Community Health Centre |
| Gauteng | Oliven Clinic |
|  | Kgabo Clinic |
|  | Phedisong Clinic |
|  | Suurman Clinic |
|  | Ramotse Clinic |
|  | Jubilee District Hospital |
|  | ODI District Hospital |
|  | Laudium Community Health Centre |
| KwaZulu-Natal | Kwamashu Community Health Centre |
|  | Stanger District Hospital |
|  | Benedictine District Hospital |
|  | Mpumelelo Clinic |
|  | Port Shepstone Hospital |
|  | Gamalakhe Community Health Centre |
|  | Itshelejuba District Hospital |
|  | Turton Community Health Centre |
|  | Northdale District Hospital |
|  | Kwadabeka Community Health Centre |
|  | East Boom Community Health Centre |
| Limpopo | Mogoto Primary Health Care Clinic |
|  | Evelyn Lekganyane Primary Health Care Clinic |
|  | Mapela Clinic |
| Mpumalanga | Mapulaleng Regional Hospital |
|  | Embhuleni Hospital |
|  | Witbank Regional Hospital |
|  | Kwaggafontein Clinic |
|  | Topsy Foundation Community Health Centre |
| Northern Cape | Galeshewe Day Hospital |
| Western Cape | Malmesbury Community Health Centre |
|  | Vredenburg Community Health Centre |
|  | Mosselbay Provincial Hospital |
